# Evidence of bidirectional relationship between type 2 diabetes and depression; a Mendelian randomization study

**DOI:** 10.1038/s41380-025-03083-0

**Published:** 2025-07-01

**Authors:** Renu Bala, Dale Handley, Alexandra Gillett, Harry Green, Jack Bowden, Andrew Wood, Inês Barroso, Cathryn M. Lewis, Jessica Tyrrell

**Affiliations:** 1 Department of Clinical and Biomedical Sciences, Faculty of Health and Life Sciences, University of Exeter, Exeter, UK; 2 Social, Genetic and Development Psychiatry Centre, Institute of Psychiatry, Psychology and Neuroscience, King’s College London, London, UK; 3 Exeter Centre of Excellence for Diabetes Research (EXCEED), University of Exeter, Exeter, UK; 4 Department of Medical and Molecular Genetics, Faculty of Life Science and Medicine, King’s College London, London, UK

**Keywords:** Depression, Genetics

## Abstract

Major depressive disorder (MDD) and type 2 diabetes (T2D) represent two global health challenges with a high degree of co-occurrence. Here, we aim to investigate the causal relationship between MDD and T2D in diverse ancestries using Mendelian randomization (MR) in GWAS summary statistic and individual level (UK Biobank (UKB)) data. We assessed the bi-directional causal relationship between: (a) MDD and T2D and (b) MDD and glycaemic biomarkers (e.g. TG:HDL-C ratio, a measure of insulin resistance, fasting glucose) in non-diabetic individuals. In UKB we also tested the role of T2D on treatment resistant depression (TRD). We used multivariable MR (MVMR) to assess the role of body mass index (BMI) in the MDD to T2D relationship. Our results demonstrated that a doubling in MDD genetic liability was associated with 1.14 higher odds of T2D (95% CI:1.09, 1.19), whilst a doubling in T2D genetic liability associated with 1.02 higher odds of MDD (95% CI:1.01, 1.03). Consistent effect estimates were observed in the UKB when stratifying by sex and suggested a role for T2D in TRD. T2D GWAS derived clusters of genetic variants highlighted the importance of specific pathways in the MDD relationship, including variants raising T2D risk via body fat (OR:1.04; 95% CI:1.02, 1.06), obesity mediated insulin resistance (OR:1.06; 95% CI:1.04, 1.09) and residual glycaemic (OR: 1.02; 95% CI:1.00, 1.04) pathways. MVMR with BMI attenuated the bidirectional relationship between MDD and T2D, particularly from MDD to T2D. Genetic liability to MDD was also associated with higher TG:HDL-C ratio in individuals without T2D (β:0.11; 95% CI:0.08, 0.14). We provide evidence of bidirectional causal association between MDD and T2D, with MDD strongly predicting insulin resistance and T2D. T2D predicted both MDD and TRD and highlighted the importance of obesity and body fat pathways in the T2D to MDD relationship.

## Introduction

Major depressive disorder (MDD) and type 2 diabetes (T2D) are two complex global health problems, that are becoming more prevalent in the population [1, 2]. It is estimated that the lifetime occurrence of MDD is 11% [3] whilst the prevalence of T2D is 9% [4]. Therefore, we would anticipate a degree of co-occurrence, however, previous studies have suggested that they occur together at twice the expected frequency [5]. This fits with several observational studies, indicating strong bidirectional correlations between T2D and MDD [6, 7]. For example, MDD was associated with a 37% higher T2D risk [5], whilst T2D was associated with a 15% higher depression risk [8]. However, these observational studies tend to be small and subject to reverse causality and unmeasured confounding. Understanding the MDD-T2D relationship is crucial as there is growing evidence that these conditions exacerbate each other, resulting in poorer outcomes. For example, individuals with T2D and MDD have higher and more variable glycated haemoglobin (HbA1c) levels, an elevated risk of T2D complications (e.g. eye problems, neuropathy and heart disease) and individuals with T2D treated with antidepressants have higher levels of HbA1c, suggesting poorer glycaemic control [9].

A genetic technique, Mendelian randomisation (MR), can be used to provide more robust insights into causation. MR uses genetic variants as instruments, and since variants are fixed from conception false inferences due to confounding and bias are reduced [10–12]. Few studies have used this approach to assess the bidirectional relationship between MDD and T2D. There is some evidence for a causal role of MDD on T2D and metabolic syndrome [13, 14]. A recent paper by Maina and colleagues, performs the most robust analyses to date, demonstrating a causal role for MDD on T2D which is partially explained by BMI, but no evidence of a bidirectional relationship [15].

Here, we build on this evidence base by (a) conducting a bi-directional MR using the most up to date summary statistics for MDD [16], T2D [17] and a range of T2D biomarkers including fasting glucose, insulin fold change (IFC), insulin sensitivity index (ISI), TG:HDL-C ratio for insulin resistance and HbA1c in individuals without T2D [18–20], (b) using subsets of T2D variants known to act via specific pathways to provide an insight into the biological mechanisms and (c) using one sample MR methods in the UK Biobank (UKB) to test for sex specific effects and to investigate more nuanced MDD and T2D phenotypes (e.g. GP record derived major depression, treatment resistant depression (TRD), severe depression, number of depression episodes, age at diagnosis of T2D). Within UKB we also performed multivariable MR (MVMR) to test the role of BMI in mediating the MDD and T2D relationship as previously suggested [15].

## Methods

### Study sample and statistical analysis used in two-sample bidirectional MR

#### Summary statistics

To assess the causal pathways between T2D and MDD, we performed 2-sample bidirectional MR using MDD as the exposure and T2D as the outcome and vice versa, using the most up to date GWAS summary statistics.

For T2D, we used GWAS summary statistics from Suzuki et al. including over 2.5 million individuals, with over 500,000 T2D cases [17]. Here, we used 1289 independent association signals at genome wide significance (GWS; *P* < 5 × 10^−8^) to instrument T2D. To tease apart potential pathways driving the association between T2D and MDD, we performed analyses using eight non-overlapping clusters of T2D variants identified in Suzuki et al. which act via specific mechanistic pathways (ST 1). The pathways include: (a) beta cell dysfunction with positive proinsulin association, (b) beta cell dysfunction with negative proinsulin association, (c) insulin resistance mediated by obesity, (d) insulin resistance mediated by lipodystrophy, (e) insulin resistance mediated by liver-lipid metabolism, (f) metabolic syndrome, (g) body fat and (h) residual glycaemic.

MDD GWAS data from Als et al. includes 371,184 individuals with MDD and 978,703 controls [16]. To instrument MDD we used the 243 genetic loci (303 independent SNPs) that were associated with MDD at GWS (ST 2).

Where possible for the bidirectional MDD-T2D relationship, we also utilised data from diverse genetic groups. For MDD to T2D, we utilised European ancestry MDD GWAS as an exposure and T2D data from (a) East Asian (EAS), (b) South Asian (SAS), (c) African (AFR) and (d) Hispanic (HISP) GWAS. We also considered data from the EAS MDD GWAS [21] as an exposure and the EAS T2D GWAS as an outcome (ST 3). Finally, multi-ancestry T2D variants were used as an exposure with EAS MDD as an outcome.

To improve our understanding of the role of MDD in T2D, we tested the bidirectional causal relationship between MDD and five T2D biomarkers. For four biomarkers (fasting glucose, HbA1c, Insulin fold change, Insulin sensitivity index) we used summary statistics from the meta-analysis of glucose and insulin related traits consortium (MAGIC) using individuals without T2D [18, 19]. We also used the triglycerides to high-density lipoprotein cholesterol (TG:HDL-C) ratio as a proxy measure of insulin resistance [20], which has previously been validated in several populations [22–24]. We selected 114 SNPs from the recent GWAS that were associated at GWS with the TG:HDL-C ratio and which were associated with at least one other IR related trait at *P* < 0.05 ensuring they are suitable proxies for insulin resistance.

All genetic variants reaching GWS in the primary GWAS and included in our analyses as exposures are summarised in ST 1–5. To assess potential pleiotropy of each variant the GWAS catalogue (https://www.ebi.ac.uk/gwas/home) was searched for known GWS hits (ST 1–4). Where an exposure SNP was unavailable, proxies were identified using the LDProxy tool (https://ldlink.nih.gov/?tab=home). Proxies were considered suitable if within 500 kb and r2 ≥ 0.8 (ST 1–5, Fig. S1).

#### Statistical analysis

Exposure SNPs and relevant proxies (ST 1–4 and Fig. S1) were extracted from the outcome GWAS summary statistics, representing the association of the outcome and exposure-trait-SNP. Published coefficients from the primary GWAS (ST 1–4) represent the exposure association with the exposure-trait-SNP. We used a custom pipeline and performed four two-sample MR methods: inverse-variance weighting (IVW), MR-Egger [10], weighted median (WM), and penalised weighted median (PWM) [11]. Here, the IVW approach represents our main analyses, with MR-Egger, WM and PWM used as sensitivity analyses to account for unidentified pleiotropy that could bias our results [25]. These methods are described in more detail in the Supplementary Methods.

When working with binary exposures we converted the causal estimates for the outcomes to represent the per unit difference in outcome per doubling of genetic liability to binary exposure (i.e. two-fold increase in the prevalence of the exposure) [26]. All betas and standard errors are multiplied by 0.693 (log_e_2) to convert the outcome to a doubling in the genetic liability of the exposure.

#### Sensitivity analyses

MR relies on several assumptions to minimize confounding and account for instrument validity (see Supplementary Methods for further details).

To assess potential confounding and pleiotropy we performed several sensitivity analyses. Firstly, we removed potentially pleiotropic variants by (a) removing variants that reached GWS with the outcome trait as well as the exposure and (b) removing variants with known associations with MDD and T2D related traits (e.g. BMI, neuroticism etc.; ST 1–4). Secondly, we performed a recently developed Bayesian framework-based MR method, MR-Horse, that is more robust to instrument invalidity (https://github.com/aj-grant/mrhorse; plus Supplementary Methods) [27]. MR-Horse tested the validity of our instruments and determined if our traditional MR results remained consistent after accounting for both correlated and uncorrelated pleiotropy. Finally, we used MRlap (https://github.com/n-mounier/MRlap; plus Supplementary Methods) [28], to account for several potential MR biases. MRlap corrects for weak instrument bias and winner’s curse, whilst accounting for sample overlap and its effect as a modifier of these biases. This was important as our T2D and MDD GWAS have a small degree of sample overlap. All MR methods have specific assumptions and limitations and whilst no-one method is perfect consistent estimates across the different methods improve the robustness of our findings.

### Study sample and statistical analysis used in one-sample MR in UK Biobank (UKB)

To enable sex stratified analyses and analyses within specific subsets of individuals we performed one-sample MR analyses in unrelated Europeans from the UKB study in up to 374,900 individuals (Supplementary Methods).

#### UKB outcomes

Several outcomes were derived in UKB including GP derived depression, treatment resistant depression (TRD), depression from the first UKB mental health questionnaire (MHQ), T2D from multiple sources, fasting glucose, HbA1c, TG:HDL-C ratio and a range of endophenotypes associated with T2D and MDD. More details on the derivation of these phenotypes are available in the Supplementary Method.

#### Statistical analysis

In UKB, all analyses follow the one-sample MR framework, where the exposure, genetic variants and the outcomes are measured in the same individuals. Individual-level data univariable MR (UVMR) analyses were carried out using two-stage least squares approach for continuous outcomes. For binary outcomes (e.g. T2D and depression), the second stage linear regression was replaced with logistic regression (otherwise known as two stage predictor substitution). MR methods are susceptible to genetic confounding due to population stratification. To address this, we adjusted for the first five genetic principal components in all analyses and only included unrelated individuals of European ancestry.

##### Software packages

All 2-sample MR analyses were performed using R version 4.1.2, whilst analyses in the UKB were performed in RStudio version 4.1.1 on the UKB RAP system.

## Results

### Our genetic instruments were robustly associated with the exposures

All our European derived instruments were robustly associated with the exposure traits with mean F-statistics of ≥23. The mean F-statistic for variants was (a) 90 for T2D (ST 1); (b) 41 for MDD (ST 2); (c) 97 for HbA1c (ST 4); (d) 127 for fasting glucose (ST 4); (e) 45 and 46 for Insulin fold change (IFC) adjusted or unadjusted for BMI; (f) 46 and 23 for modified Stumvoll insulin sensitivity index (ISI) adjusted for or unadjusted for BMI; and (g) 168 for TG:HDL-C ratio.

### Genetically instrumented T2D was associated with higher odds of MDD

**Fig. 1:**
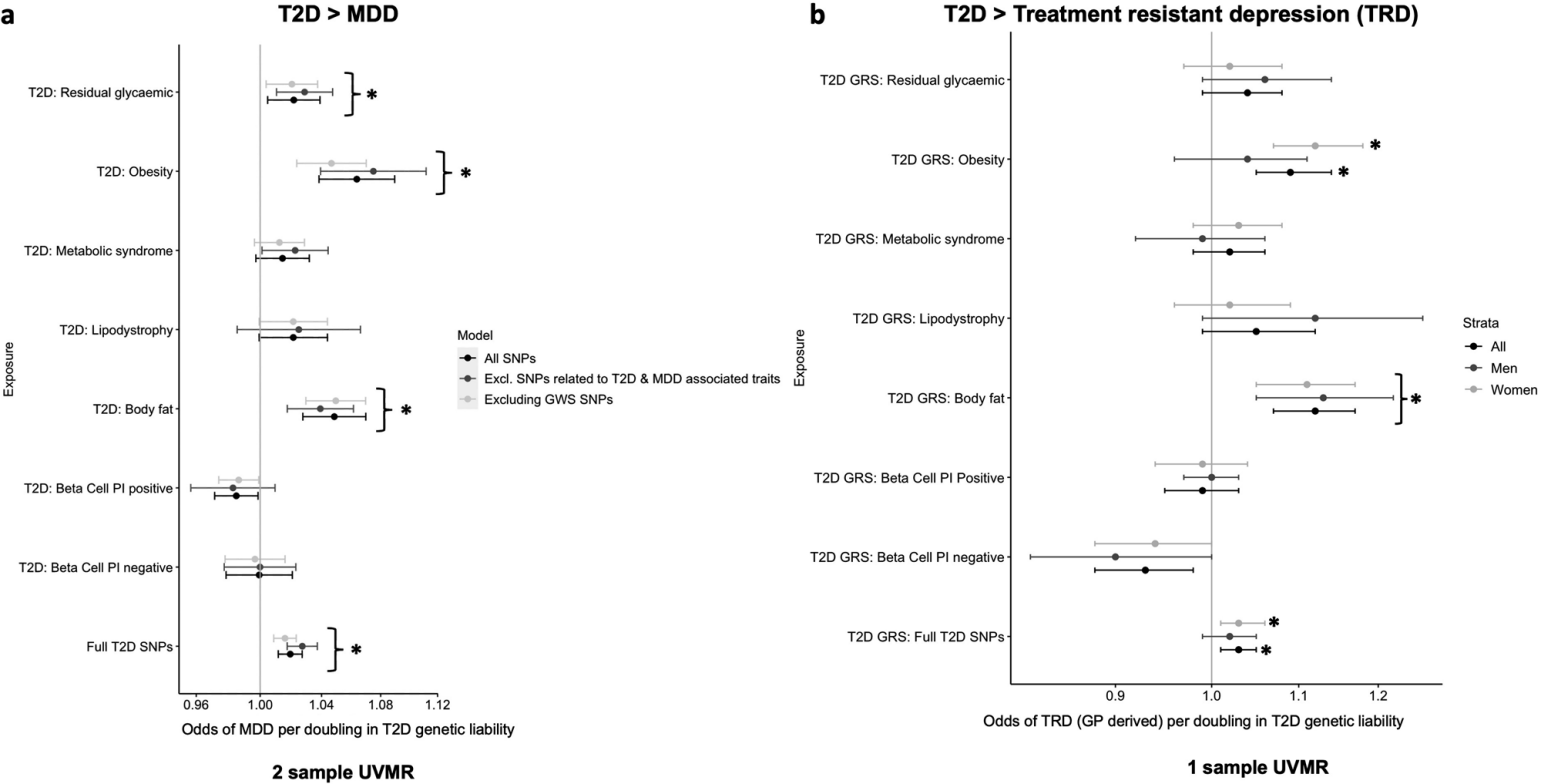
Summary of 1-sample and 2-sample UVMR results. **a** T2D exposure > MDD outcome: 2-sample UVMR (IVW estimates) showing the odds of MDD per doubling in the genetic liability of T2D. The figure includes all T2D SNPs from the GWAS (Full T2D SNPs) as well as the 7 subsets of SNPs hypothesised to act via specific pathways (Note: subset of liver lipid metabolism has not been shown in the figure due to large confidence intervals), **b** T2D exposure > Treatment resistant depression (TRD) outcome: 1-sample UVMR in UK-biobank showing the odds of TRD (GP records) per doubling in the genetic risk of T2D using genetic risk score (GRS) for all T2D SNPs and its 8 subsets. The odds of TRD were significantly higher using full T2D GRS and in body fat and obesity subset, while lower odds for MDD in Beta cell PI negative subset. *p value <0.05 (Abbreviations used: GWS genome wide significance, SNP single nucleotide polymorphism, MDD major depressive disorder, IVW inverse variance weighed, PI proinsulin, neg negative, pos positive, UVMR univariable Mendelian randomization, TRD treatment resistant depression, GRS genetic risk score, GP general practitioner) (Note: All SNPs: includes all T2D variants at GWS in the primary T2D GWAS as an exposure; excluding GWS SNPs: removes any variants that are associated at GWS with the outcome (i.e. MDD); excluding SNPs related to T2D & MDD associated traits: searching the GWAS catalogue for GWS associations involving the exposure variant or nearby variants in LD, to identify and removing the variants that are associated with related traits (e.g. BMI, body fat, anxiety, neuroticism etc.) to minimize any potential pleiotropic effect).

2-sample MR using all 1192 available genetic variants provided evidence that a doubling in T2D genetic liability associated with 1.02 (95%CI: 1.01, 1.03, *P* = *8.91*E–7) times higher odds of depression (Table 1, Fig. 1a). There was evidence of horizontal pleiotropy from MR-Egger (*P* = 2.6E–8; Table 1). Removing 226 variants with known GWS associations with MDD and T2D associated traits strengthened our results (OR: 1.03 [95%CI: 1.02, 1.04], *P* = *5.5*E–08; ST 6). Findings were consistent with the more pleiotropy robust methods (ST 6, 7) and MRlap provided no evidence of bias from sample overlap, weak instrument or Winner’s curse (p-difference: 0.59) (ST 8).

**Table 1:** 2-sample MR results representing the odds of outcome trait per doubling in exposure trait genetic liability.

Exposure trait	Outcome trait	Beta/OR (95% CI) representing change in outcome per doubling in T2D or MDD genetic liabilty (IVW)	*P* Value (IVW)	Evidence of horizontal pleiotropy from MR egger	Number of SNPs
Full T2D SNPs	MDD	1.019 (1.011, 1.027)	**1.0E–06**	**Yes,** ***p*** = **2.12E–08**	1192
T2D: Beta Cell PI negative	MDD	0.999 (0.978, 1.020)	9.6E–01	No, *p* = 0.127	83
T2D: Beta Cell PI positive	MDD	0.985 (0.971, 0.998)	**3.3E–02**	No, *p* = 0.962	86
T2D: Body fat	MDD	1.048 (1.027, 1.069)	**6.4E–06**	**Yes,** ***p*** = **0.025**	243
T2D: Lipodystrophy	MDD	1.021 (0.999, 1.043)	6.3E–02	No, *p* = 0.965	45
T2D: Metabolic syndrome	MDD	1.014 (0.997, 1.031)	1.1E–01	No, *p* = 0.450	160
T2D: Obesity	MDD	1.064 (1.038, 1.089)	**1.1E–06**	No, *p* = 0.847	233
T2D: Residual glycaemic	MDD	1.022 (1.004, 1.038)	**1.2E–02**	**Yes,** ***p*** = **0.004**	339
T2D: liver lipid metabolism	MDD	0.924 (0.857, 0.996)	1.8E–01	**Yes,** ***p*** = **0.0007**	3
MDD	T2D	1.139 (1.093, 1.187)	**2.33E–09**	No, *p* = 0.88	293
MDD	Fasting Glucose	0.003 (−0.007, 0.013)	0.541	No, *p* = 0.96	303
MDD	HbA1c	0.004 (−0.003, 0.011)	0.302	No, *p* = 0.13	303
MDD	TG:HDL-C ratio	0.11 (0.075,0.137)	**7.30E–11**	No, *p* = 1.00	302
MDD	Insulin Fold Change (adj BMI)	−0.010 (−0.039, 0.018)	0.486	No, *p* = 0.8	303
MDD	Insulin Fold Change (not adj BMI)	−0.016 (−0.044, 0.013)	0.292	No, *p* = 0.63	303
MDD	Modified Stumvoll ISI (adj BMI)	−0.003 (−0.033, 0.026)	0.819	No, *p* = 0.25	303
MDD	Modified Stumvoll ISI (not adj BMI)	−0.015 (−0.044, 0.013)	0.292	No, *p* = 0.63	303
HbA1c	MDD	0.992 (0.915, 1.076)	0.854	**Yes,** ***p*** = **0.01**	85
Fasting glucose	MDD	0.946 (0.886, 1.010)	0.105	No, *p* = 0.31	78
TG:HDL-C ratio	MDD	1.02 (0.99, 1.05)	0.089	No, *p* = 0.67	114
Insulin Fold Change (adj BMI)	MDD	1.017 (0.937, 1.103)	0.711	No, *p* = 0.86	5
Insulin Fold Change (not adj BMI)	MDD	1.017 (0.937, 1.102)	0.710	No, *p* = 0.86	5
Modified Stumvoll ISI (adj BMI)	MDD	1.011 (0.96, 1.063)	0.695	No, *p* = 0.35	8
Modified Stumvoll ISI (no adj BMI)	MDD	1.022 (0.951, 1.097)	0.563	No, *p* = 0.78	8

*T2D* type 2 Diabetes; *SNPs* single nucleotide polymorphism; *PI* proinsulin; *GWS* genome wide significance; *MDD* major depressive disorders; *SE* standard error; *MR* mendelian randomization; *CI* confidence interval; *IVW* inverse variance weighed; *OR* odds ratio; *adj BMI* adjusted body mass index; *HbA1c* glycated haemoglobin; *ISI* insulin sensitivity index; *TG:HDL-C ratio* triglycerides to high-density lipoprotein cholesterol ratio.

Stratifying the T2D genetic variants into specific clusters provided evidence that the T2D and MDD association is predominantly driven by variants from the obesity mediated insulin resistance and body fat pathways (Fig. 1a). A doubling in T2D genetic liability using variants that increase T2D risk via obesity mediated insulin resistance associated with 1.06 higher odds of MDD (95%CI: 1.04, 1.09; *P* = 1.09E–6; Table 1, Fig. 1a). Similarly, a doubling in T2D genetic liability using variants that increase T2D risk via body fat pathways associated with a 1.05 (95%CI: 1.03, 1.07; *P* = 6.37E–06) higher odds of MDD (Table 1, Fig. 1a). Findings were consistent with (a) more pleiotropy robust methods (ST 6, 7) and (b) removal of variants associated at GWS with MDD and variants associated with MDD or T2D related traits at GWS (ST 6).

Genetic variants associated with higher T2D genetic liability via residual glycaemic pathways associated with higher odds of MDD (OR: 1.02; 95%CI: 1.00, 1.04; *P* = 0.016). Results were consistent upon removal of potentially pleiotropic loci (ST 6) with some evidence of horizontal pleiotropy (*P* = 0.004).

There was less consistent evidence for T2D variants acting via alternative pathways (Table 1). Variants associated with higher T2D risk via beta cell dysfunction with positive pro-insulin association were nominally associated with lower odds of MDD (OR: 0.98; 95%CI: 0.97, 1.00; *P* = 0.034; Table 1). In contrast T2D variants acting via metabolic syndrome pathways may associate with higher odds of MDD, especially when excluding variants known to be pleiotropic (Table 1 and ST 6). There was no evidence for an association between T2D variants that act via lipodystrophy or beta cell dysfunction with negative pro-insulin association and MDD (Table 1, ST 6). The liver-lipid metabolism T2D cluster only had three variants and therefore our findings were inconclusive.

### Findings were consistent in the UKB 1-sample analyses with higher genetic risk of T2D predicting depression

We demonstrated a doubling in T2D genetic risk was associated with 1.02 (95% CI: 1.01, 1.04, *P* = 0.002) higher odds of MHQ derived major depression (ST 9), with similar results in a larger subset with GP derived major depression (ST 9 and Fig. S2).

A doubling in the genetic liability of T2D predicted 1.06 higher odds of TRD (95%CI: 1.02, 1.11; ST 9, Fig. 1b). There was no genetic evidence for an association of T2D with (a) number of depression episodes and (b) depression duration in years (ST 9).

When using the 8 pathway specific subsets of T2D variants, we observed similar results to the 2-sample MR, with the strongest evidence of an association between T2D and MDD for T2D variants acting via (a) body fat pathways and (b) obesity mediated insulin resistance pathways (ST 9). These subsets were also associated with TRD (ST 9, Fig. 1b). There was also tentative evidence that the body fat cluster of T2D variants predicted 1.18 times higher odds of having TRD compared to non-treatment resistant MDD (95%CI: 1.04, 1.35; *P* = 0.012) (ST 9).

### Genetically instrumented T2D was not associated with higher odds of MDD in East Asians

We found no association of higher genetic liability to T2D with increased risk of MDD in individuals of EAS ancestry (OR: 1.00, 95% CI: 0.99, 1.00; *P* = 0.52), with similar results across different T2D subsets and pleiotropy robust methods (Table 2, ST 1, 10).

**Table 2:** 2-sample MR results demonstrates the odds of outcome trait in different ancestries.

Exposure trait	Exposure trait ancestry	Outcome trait	Outcome trait ancestry	Model	OR (95%CI) of outcome trait per doubling in exposure trait genetic liability (IVW)	*p* Value (IVW)	Evidence of horizontal pleiotropy from MR egger	Number of SNPs
Full T2D SNPs	EUR+ African+ HISP+EAS+SAS	MDD	EAS	All SNPs	1.00 (0.99, 1.00)	0.53	**Yes,** ***p*** = **0.03**	1034
T2D: Beta Cell PI neg	EUR+ African+ HISP+EAS+SAS	MDD	EAS	All SNPs	1.012 (0.93, 1.092)	0.75	No, *p* = 0.85	70
T2D: Beta Cell PI pos	EUR+ African+ HISP+EAS+SAS	MDD	EAS	All SNPs	0.98 (0.92, 1.04)	0.57	No, *p* = 0.81	60
T2D: Body fat	EUR+ African+ HISP+EAS+SAS	MDD	EAS	All SNPs	1.00 (0.99, 1.00)	0.09	No, *p* = 0.07	214
T2D: Lipodystrophy	EUR+ African+ HISP+EAS+SAS	MDD	EAS	All SNPs	0.93 (0.81, 1.06)	0.31	No, *p* = 0.44	36
T2D: Metabolic syndrome	EUR+ African+ HISP+EAS+SAS	MDD	EAS	All SNPs	1.09 (1.01, 1.17)	**0.01**	No, *p* = 0.28	139
T2D: Obesity	EUR+ African+ HISP+EAS+SAS	MDD	EAS	All SNPs	0.99 (0.99, 1.00)	0.66	**Yes,** ***p*** = **0.04**	207
T2D: Residual glycaemic	EUR+ African+ HISP+EAS+SAS	MDD	EAS	All SNPs	0.99 (0.99, 1.00)	0.97	No, *p* = 0.34	305
T2D: liver lipid metabolism	EUR+ African+ HISP+EAS+SAS	MDD	EAS	All SNPs	0.56 (0.38, 0.81)	0.09	No, *p* = 0.89	3
MDD	European	T2D	EAS	All SNPs	1.0469 (0.999, 1.095)	0.053	No, *p* = 0.59	293
MDD	European	T2D	SAS	All SNPs	1.093 (1.018, 1.172)	**0.014**	No, *p* = 0.26	293
MDD	European	T2D	AFA	All SNPs	0.979 (0.929, 1.032)	0.447	No, *p* = 0.89	293
MDD	European	T2D	HISP	All SNPs	1.115 (1.044, 1.190)	**0.001**	No, *p* = 0.76	293
MDD	EAS	T2D	EAS	All SNPs^a^	0.978 (0.65–1.45)	0.92	**Yes,** ***p*** = **0.007**	5

*T2D* type 2 diabetes; *SNPs* single nucleotide polymorphism; *PI* proinsulin; *MDD* major depressive disorders; *SE* standard error; *MR* mendelian randomization; *CI* confidence interval; *IVW* inverse variance weighed; *OR* odds ratio; *EAS* east asian; *EUR* european; *SAS* south asian; *HISP* hispanic; *GWS* genome wide significance.

^a^SNPs with and without GWS in exposure GWAS.

### Genetically instrumented depression was associated with higher odds of T2D

**Fig. 2:**
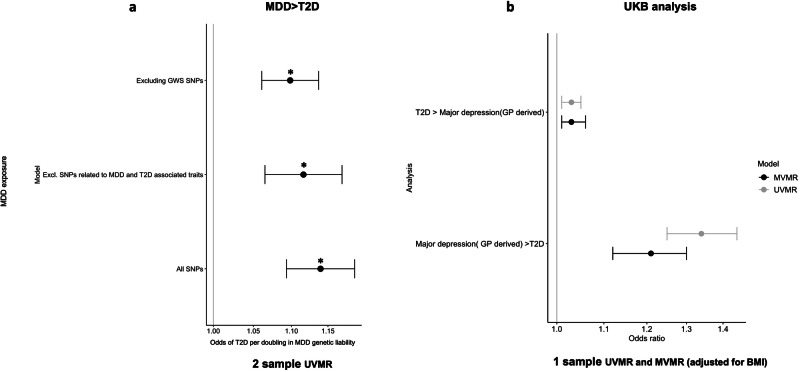
Findings from 1-sample UVMR & MVMR and 2-sample UVMR analysis. **a** MDD exposure > T2D outcome: 2-sample UVMR (IVW estimates) showing the odds ratios for T2D per doubling in MDD genetic liability. The different models presented represent including all MDD variants, excluding those at GWS with T2D and excluding all SNPs with known associations with MDD/T2D related traits, **b** 1-sample UVMR and MVMR (adjusted for BMI) in UK-biobank in all individuals. *p value <0.05 (Abbreviations used: GWS genome wide significance, SNP single nucleotide polymorphism, MDD Major depressive disorder, IVW Inverse variance weighed, UVMR univariable Mendelian randomization, MVMR multivariable Mendelian randomization, BMI body mass index, MHQ mental health questionnaire, GP general practitioner, GRS genetic risk score) (Note: All SNPs: includes all MDD variants at GWS in the primary MDD GWAS as an exposure; excluding GWS SNPs: removes any variants that are associated at GWS with the outcome (i.e. T2D); excluding SNPs related to MDD & T2D associated traits: searching the GWAS catalogue for GWS associations involving the exposure variant or nearby variants in LD, to identify and removing the variants that are associated with related traits (e.g. BMI, body fat, anxiety, neuroticism etc.) to minimize any potential pleiotropic effect).

A doubling in the genetic liability of MDD, using 293 variants, was associated with 1.14 (95%CI: 1.09, 1.19; *P* = 2.33E–09) higher odds of T2D (Table 1). Our findings remained consistent after (a) using more pleiotropy robust methods, (b) removing variants associated with T2D at GWS and (c) removing variants associated at GWS with either MDD or T2D related traits (ST 7, 11, Fig. 2a). MR Lap suggested some bias as a result of sample overlap, Winner’s curse or weak instruments, however when using the corrected estimates, a doubling in MDD genetic liability still predicted T2D (OR: 1.10 (95%CI: 1.06, 1.13)) (ST 8).

### Genetically instrumented MDD using European ancestry weights was associated with higher odds of T2D in individuals of South Asian, and Hispanic ancestries

A doubling of the genetic liability to depression was associated with higher odds of T2D in individuals of (a) SAS and (b) HISP ancestries (Table 2). Results were consistent across pleiotropy robust methods (ST 7, 12).

There was no association of MDD with the risk of T2D in individuals of EAS and AFR ancestry (Table 2, ST 7, 12). Further, using the 5 instruments (mean F-statistics:17) identified in the EAS MDD GWAS, there was no evidence of an association between MDD and T2D, but this is likely due to lack of power (Table 2, ST 3, 12).

### Findings were consistent in UKB, with a higher genetic liability of MDD predicting T2D in both men and women

Findings were similar using 1-sample MR approaches in UKB where a doubling in MDD genetic liability (MHQ derived) was associated with 1.29 higher odds of T2D (95%CI: 1.17, 1.42; *P* = 1E–7) with similar results in males and females alone (ST 9, Fig. S3). Results were consistent when using the GP derived depression metric for the first step of the model (OR: 1.34 (95% CI:1.25, 1.44); *P* = 3E–16) (ST 9, Fig. S3).

A doubling in genetic liability to MDD was also associated with 0.67 (95% CI: 0.19, 1.15) years earlier T2D diagnosis (ST 9).

### MVMR suggests BMI partially explains the bi-directional relationship between T2D and MDD

Using MVMR, we provide evidence that BMI partially explains the T2D-MDD relationship, with adjustment for BMI attenuating the associations between T2D and MDD towards the null in UKB. For example, the association between T2D and MHQ derived MDD attenuates to the null (OR: 1.01; 95% CI: 1.00, 1.03; *P* = 0.07), whilst a nominal association remains with GP derived MDD (OR: 1.03; 95% CI: 1.01, 1.06; *P* = 0.02) (ST 9, Fig. 2b).

Interestingly, using the body fat subset of T2D variants as an exposure in MVMR, adjusting for BMI slightly strengthened the association with MDD (GP derived) compared to the univariable model (ST 9). These results suggest that higher genetic susceptibility to T2D may act via body fat pathways to increase the risk of depression and is not mediated via BMI.

Depression remains a strong causal predictor for T2D when adjusting for BMI. A doubling in the genetic liability of MDD remained associated with higher odds of T2D using either the MHQ or GP derived depression metric (OR: 1.18 (95% CI: 1.07, 1.30) and 1.21 (95% CI: 1.12, 1.30) respectively (ST 9, Fig. 2b).

### Genetically instrumented depression was associated with higher TG:HDL-C ratio but not with other glycaemic biomarkers in individuals without T2D

**Fig. 3:**
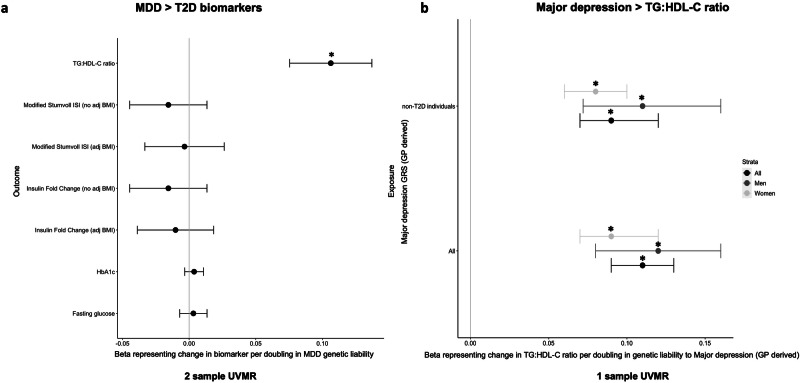
Summary of results from 1-sample and 2-sample UVMR analysis. **a** MDD exposure > T2D biomarkers outcome: 2-sample UVMR results (IVW) representing the change in the T2D biomarker per doubling in MDD genetic liability, **b** major depression exposure > TG:HDL-C ratio outcome: 1-sample UVMR in UK-biobank shows increase in TG:HDL-C ratio in all and non-T2D individuals in sex stratified analysis per doubling in genetic liability to major depression (GP derived) GRS. *p value <0.05 (Abbreviations used: MDD major depressive disorder, IVW inverse variance weighed, ISI insulin sensitivity index, BMI body mass index, adj adjusted, HbA1c glycated haemoglobin, TG-HDL-C triglycerides to high density lipoprotein cholesterol ratio, UVMR univariable Mendelian randomization, T2D type 2 diabetes, GP general practitioner, GRS genetic risk score, TG-HDL-C Triglycerides to high density lipoprotein cholesterol ratio).

A doubling in MDD genetic liability was strongly associated with higher TG:HDL-C ratio (β: 0.11; 95% CI:0.075, 0.14; *P* = 7.3E–11) with no evidence of horizontal pleiotropy and consistent results across various sensitivity methods (Table 1, ST 7, 11, Fig. 3a).

Findings for TG:HDL-C ratio were consistent in UKB, where a doubling in genetic liability to depression (GP derived) was associated with higher TG:HDL-C ratio in all (β:0.11; 95% CI: 0.09, 0.13; *P* < 2E–16) and non-T2D individuals (β:0.09; 95% CI: 0.07, 0.12; *P* = 3E–16) (ST 9, Fig. 3b). Findings were consistent with MHQ derived depression phenotype and in sex stratified analysis (ST 9). When BMI was included in the MVMR model, depression remained a strong predictor of TG:HDL-C ratio in all individuals and those without T2D (ST 9).

The primary IVW analysis provided no evidence for an association of MDD with higher fasting glucose, fasting insulin and HbA1c levels in individuals without T2D (Table 1, ST 11, Fig. 3a). However, with the removal of GWS variants and using more pleiotropy robust methods (WM and PWM) there was some evidence that a doubling in MDD genetic liability was associated with higher HbA1c levels (ST 11).

There was limited genetic evidence of MDD associating with other glycaemic biomarkers in individuals without T2D in UKB. For example, a doubling in genetic liability to depression was associated with higher HbA1c and fasting glucose levels in all individuals, but not when excluding those with a T2D diagnosis (ST 9).

### There was limited evidence that glycaemic biomarkers other than TG:HDL-C ratio were associated with depression or associated phenotypes

**Fig. 4:**
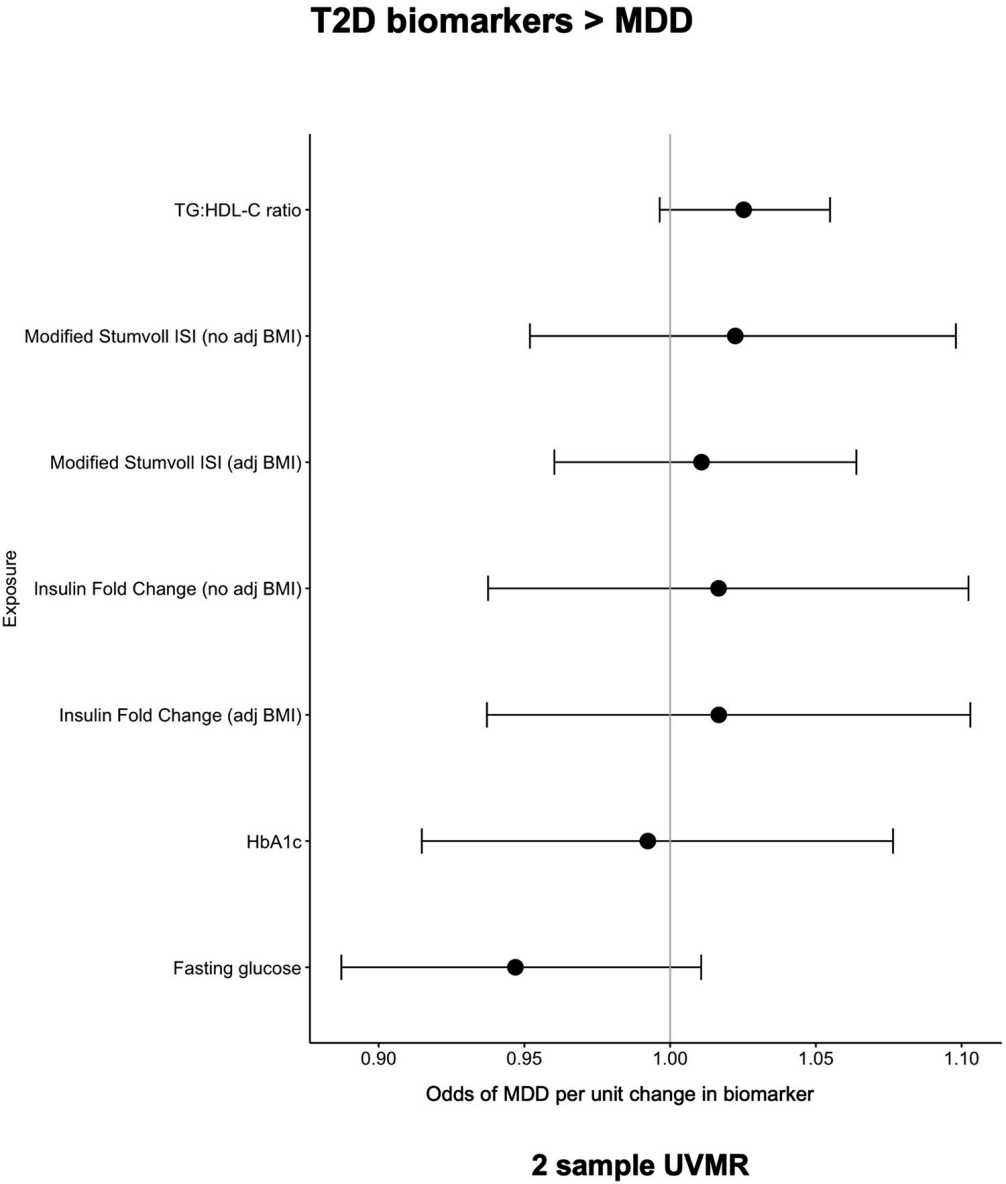
2-sample MR results (IVW) representing the odds of MDD per unit change in genetically instrumented biomarkers. T2D biomarkers did not predict the risk of MDD (MDD major depressive disorder, IVW inverse variance weighed, ISI insulin sensitivity index, BMI body mass index, adj adjusted, HbA1c glycated haemoglobin, TG-HDL-C triglycerides to high density lipoprotein cholesterol ratio).

2-sample MR analysis, provided limited evidence for a role of fasting glucose, IFC, ISI, TG:HDL-C ratio and glycated haemoglobin in depression (Table 1, Fig. 4). There was some evidence that higher genetically instrumented HbA1c levels associated with higher odds of depression, but only in MR Egger estimates; which also indicated our IVW analyses may be biased by horizontal pleiotropy (*P* = 0.01) (ST 13).

In UKB, the HbA1c estimates were directionally consistent, but the confidence intervals crossed the null, whilst genetically instrumented higher fasting glucose was associated with 1.34 higher odds of GP derived major depression in individuals without T2D (ST 9). Further, in UKB genetically instrumented higher HbA1c and fasting glucose levels were not associated with the risk of (a) TRD, (b) number of depression episodes and (c) severe depression in either men or women (with and without T2D) (ST 9).

In 2-sample MR there was limited evidence for a role of the TG:HDL ratio in MDD (Table 1). However, in UKB a genetically instrumented higher TG:HDL ratio was associated with 1.12 higher odds of MDD (GP records) (95%CI: 1.03, 1.22; *P* = 0.006) in all individuals and the association remained (a) in individuals without T2D (ST 9, Fig. S4 and (b) when adjusting for BMI in MVMR models (all individuals OR:1.13; 95% CI: 1.04, 1.11; *P* = 0.003 and non-T2D individuals OR:1.12; 95% CI: 1.01, 1.22; *P* = 0.049; ST 9).

## Discussion

We performed a comprehensive genetic analysis of the relationships between depression and T2D using the largest available studies. We found robust evidence of a bidirectional causal association between depression and T2D.

Our MR analyses build on the epidemiological findings of a bidirectional relationship between depression and T2D, providing evidence of a causal relationship in both directions. We build on the work by Maina and colleagues [15] who provided evidence of a causal relationship from depression to T2D. Here, using a larger MDD instrument from the most recent depression GWAS, we provide further robust evidence for a role of depression in causing T2D in European individuals and for the first time those of Hispanic and South Asian ancestry. We also demonstrated that depression liability predicts a higher TG:HDL-C ratio, an indirect measure of insulin resistance. Using 1-sample methods in the UKB we were able to confirm similar effects in males and females for depression to T2D and the TG:HDL-C ratio and confirm a higher genetic liability to depression predicted insulin resistance even after adjusting for BMI in all and non-diabetic individuals. UKB also provided some evidence that a higher depression genetic liability was associated with higher HbA1c in all individuals and higher fasting glucose in men, although these associations attenuated to the null in individuals without T2D. Depression was also associated with an earlier age at diagnosis of T2D in all individuals within UKB. Additionally, genetic liability to higher fasting glucose and TG:HDL-C levels predicted depression in non-diabetic individuals in UKB. Our findings fit with the known pathophysiology, with depression usually onsetting in adolescence or early adulthood [29], whilst diabetes develops later in life [30]. In addition to higher susceptibility of depression in adolescents, depression symptoms in children and adolescents are associated with increased [31] and worsening insulin resistance over time irrespective of BMI [32]. It has been shown that depression negatively affects a person’s nutritional and lifestyle choices (e.g. increased rates of smoking, poorer diet, less physical activity) and use of antidepressants may further contribute to obesity and insulin resistance, which increases an individual’s risk of T2D [33, 34]. Using MVMR we confirmed findings by Maina and colleagues that BMI partially mediates the depression to T2D relationship. Further we provided evidence that the MDD to TG:HDL-C ratio relationship persists when accounting for BMI, suggesting the link between MDD and T2D could be partially explained by insulin resistance independently of BMI.

Using the largest available T2D GWAS we provide the first evidence that T2D causes depression in Europeans. Whilst the effect sizes were small, this contrasts to the Maina et al. study which found no evidence of bidirectional causal pathways using a smaller T2D GWAS. This fits with observational data and MR studies in East Asians [35] that suggests both T2D and insulin resistance are risk factors for depression [5, 36]. Several pathways could explain this finding. For example, both hypo- and hyper-glycaemia can have major effects on brain function in areas of cognition and mood. Animal models demonstrate that diabetes negatively affects hippocampal integrity and neurogenesis [37], which may contribute via neuroplasticity to mood symptoms. Further, individuals with T2D are at an increased risk of metabolic problems including dyslipidaemia and hypertension which may increase depression risk. Within UKB we also provided evidence that a higher genetic liability to T2D predicts a more severe subtype of depression: treatment resistant depression (TRD). Here, individuals do not respond to antidepressants and switch drug classes regularly. This fits with research suggesting individuals with T2D respond poorly to antidepressants [38] with high rates of depression recurrence [39]. Studies have also shown contradicting results of antidepressants on glycaemic control [40].

We utilised subsets of T2D variants to further understand the mechanistic pathways that may drive the causal pathway from T2D to depression. The strongest associations were observed for genetic variant clusters that increase an individual’s T2D risk via (a) obesity mediated insulin resistance and (b) body fat. These clusters were also associated with TRD. This highlights the potential importance of obesity and body fat in the T2D to depression relationship. This builds on work by ourselves and others, demonstrating that higher BMI is a key risk factor for depression in Europeans [41, 42]. These subset analyses complement our MVMR analysis with BMI, which attenuates the association, further highlighting the importance of body fat and obesity in the T2D-depression relationship. The role of BMI may reflect the adverse consequences of higher BMI in relation to T2D (i.e. more complications and poorer health) or it could reflect the sociocultural consequences of higher BMI, including increased stigma [41, 42]. We have previously highlighted the association between genetic variants that are associated with increased obesity but a metabolically healthier profile and a higher risk of depression, suggesting the relationship is not purely explained by poorer health [42]. Further, the obesity mediated insulin resistance subset of variants add weight to a role for insulin resistance pathways in the T2D-MDD relationship.

In addition to phenotypic associations of T2D subset variants (described in the Suzuki et al. paper), multiple gene loci within the obesity and body fat subsets belong to biological pathways linked to depressive symptoms in human and animal studies, including the gonadotropin releasing hormone receptor (*PPARG, PPP3CA, CAMK2B, NFATC2, ITGA1, GNAS, EP300, MAP3K11* etc.), p53 (*HMGB1, MDM4, SIN3A, EP300*), Wnt signalling (*CTBP1, NFATC1, PCDH15, TLE4*) and angiogenesis (*VEGFA, FGFR1, GRB14, FRS2*) pathways [43–46]. Additionally, dysregulation of neuroendocrine and neuroinflammatory processes has been observed in depression, however the exact molecular mechanisms involved remain unknown [47].

There was also evidence for a potential role of the T2D genetic variants assigned to the residual glycaemic cluster. This cluster associates with higher fasting glucose, waist hip ratio (WHR) and blood pressure. Previous studies have indicated a role for these factors in predicting depression [48–51], although here we provided no genetic evidence for a role of fasting glucose in predicting MDD. Based on the results from the residual glycaemic cluster, future studies should investigate the potential mediation of the T2D to depression relationship by other obesity metrics (e.g. WHR and waist circumference) and fasting glucose and blood pressure.

We tested the role of glycaemic traits on depression and vice versa. Genetic liability to MDD was associated with higher insulin resistance (TG:HDL-C ratio) in both 1- and 2-sample MR analyses but there was only evidence of association in the opposite direction in UKB 1-sample analysis. In UKB the bidirectional relationship between insulin resistance and MDD remained even when accounting for BMI.

For the other glycaemic traits, when including all MDD variants in 2-sample analysis there was no clear evidence of an association. However, when we excluded GWS variants, the more pleiotropic robust methods provided some evidence that a higher genetic liability to depression was associated with higher HbA1c levels. In UKB a higher genetic liability to depression predicted both HbA1c and fasting glucose levels in all individuals, but the uncertainty around the estimate crossed the null in individuals without T2D. This fits with observational analyses demonstrating the association between depression and fasting glucose, insulin resistance and HbA1c [52, 53]. Our findings suggests that in addition to poor glycaemic control, insulin resistance might partially explain the relationship between MDD and T2D. This fits with previous studies that have demonstrated dysregulation of insulin signalling pathways associates with neuropsychiatry disorders [54] and mouse models where insulin receptor knockouts in astrocytes result in anxiety and depression-like symptoms [55]. These findings suggest the potential benefit of monitoring and managing both depression and insulin resistance in clinical practice. Monitoring of the TG: HDL-C ratio in individuals with depression may help in early intervention and prevention of the subsequent development of T2D.

UKB enabled us to perform sex stratified analyses to test if the causal relationship between T2D and depression is consistent. This is important as it is well documented that depression is more common in women [56], whilst T2D is more common in men [57]. Effect estimates were similar for the odds of depression per doubling in T2D genetic risk, but the uncertainty around the estimate was larger in men, especially when using the variants that increase T2D risk via obesity mediated insulin resistance. This increased uncertainty may be attributed to reduced statistical power due to a smaller number of depression cases in men or could reflect a genuine sex-specific difference.

This study had many strengths. Firstly, we used the largest available GWAS data of T2D and MDD, as well as data from MAGIC on glycaemic traits. Secondly, we were able to use UKB individual level data, including validated definitions of mental health outcomes [58, 59], this, unlike results from meta-analysis, gives us homogeneity of definitions, an issue that is particularly important in mental health research. Thirdly, we used several MR methods to account for MR biases such as pleiotropy, sample overlap and Winner’s curse [27, 28]. Finally, we attempted to address our questions in several different ancestries where the data was available with generally consistent findings for MDD to T2D across ancestries.

### Limitations

We acknowledge several limitations. First, there is a lack of transferability (<50%) between MDD GWAS hits from individuals of European ancestry to other ancestries [60]. This means that whilst we have tested MDD to T2D in several different ancestries further analyses should focus on using ancestry specific MDD variants (although currently this lacks power). Second, data is not available for all ancestries of interest. For example, there is only currently GWAS summary statistic data for MDD in EAS and EUR. This is particularly important for MDD as it is heterogenous in nature and presentations may vary across cultures, potentially reducing the reliability of European derived variants in other ancestries. However, the recent Psychiatric Genomics Consortium MDD GWAS [61] has provided some insights into it. Firstly, structural equation modelling of source of MDD cases showed high levels of agreement on genetic contributions across clinical recruitment, electronic health records, questionnaire data and self-report of diagnosis. Secondly, polygenic scores generated from European-ancestry GWAS summary statistics provided some prediction in all ancestry groups tested, but this was minimal in African studies. Further research is required to validate these findings across depression subtypes and in diverse ancestries to better understand the underlying mechanisms. Third, our UKB findings are not generalisable to the population as UKB is not population representative, with overrepresentation of females and higher SES individuals [62] and there is participation bias within the MHQ [63]. Fourth, within UKB MDD could be defined from multiple sources, we used two different depression definitions derived from the MHQ and GP records, whilst our results were generally consistent, they tended to be stronger with GP derived depression. This may reflect the GP derived metric more accurately capturing MDD than MHQ data. Additionally, within UKB there could be undiagnosed T2D cases among the control population, which could bias our results to the null. However, we performed sensitivity analyses excluding individuals with a HbA1c > 6.5% and observed no significant changes in our findings (data not shown). Fifth, no MR methods are perfect, with each having assumptions and limitations. Here, we attempted to assess several potential biases demonstrating consistent findings using different MR methods (e.g. MRLap and MR Horse), therefore improving the reliability of our findings. Finally, we did not set any specific threshold to account for multiple testing because our mental health phenotypes are correlated, i.e. not truly independent from each other, and therefore corrections such as Bonferroni’s are too conservative. We, instead, report confidence intervals for all our estimates.

## Conclusion

In conclusion, we have provided strong evidence of bidirectional causal association between MDD and T2D, and a higher genetic risk to MDD predicts insulin resistance (increased TG:HDL-C ratio) and higher HbA1c levels in individuals without T2D. This adds to the evidence base that MDD might result in poorer health habits and/or metabolic disturbances including insulin resistance, which increases T2D risk. There was also evidence that T2D predicts depression, with some evidence of the importance of obesity mediated insulin resistance and body fat in driving this relationship. Our study highlights the need for further research to understand this complex relationship in more detail and to determine if targeted treatment plans are required for individuals with T2D and depression.

## Supplementary information

Suplementary figures

Suplementary tables (1-13)

## Data Availability

• The T2D summary statistics are available here: http://diagram-consortium.org/downloads.html. • The glycaemic traits summary statistics are available here:  ◦ http://magicinvestigators.org/downloads/.   ◦ https://www.ebi.ac.uk/gwas/studies/GCST90295949. • The MDD summary statistics are available here: https://ipsych.dk/en/research/downloads/. • UKB data are available to any bona fide researcher following application: https://www.ukbiobank.ac.uk/enable-your-research/apply-for-access.

## Code availability

All code used for analysis can be found as follows: MR analysis: The 2 sample MR and 1-sample MR analysis has been done using the modified code available on https://github.com/MRCIEU/TwoSampleMR. MR Lap: https://github.com/n-mounier/MRlap. MR Horse: https://github.com/aj-grant/mrhorse. Genetic risk score: https://github.com/hdg204/GRS-Nexus.
