# Supplementary material for: Evidence of bidirectional relationship between type 2 diabetes and depression; a Mendelian randomization study
Source: Mol Psychiatry. 2025 Jul 1;30(11):5013–23. doi: 10.1038/s41380-025-03083-0 (PMC12532585; doi:10.1038/s41380-025-03083-0)
Supplement: Supplementary file 1 — Suplementary figures [file 41380_2025_3083_MOESM1_ESM.docx]

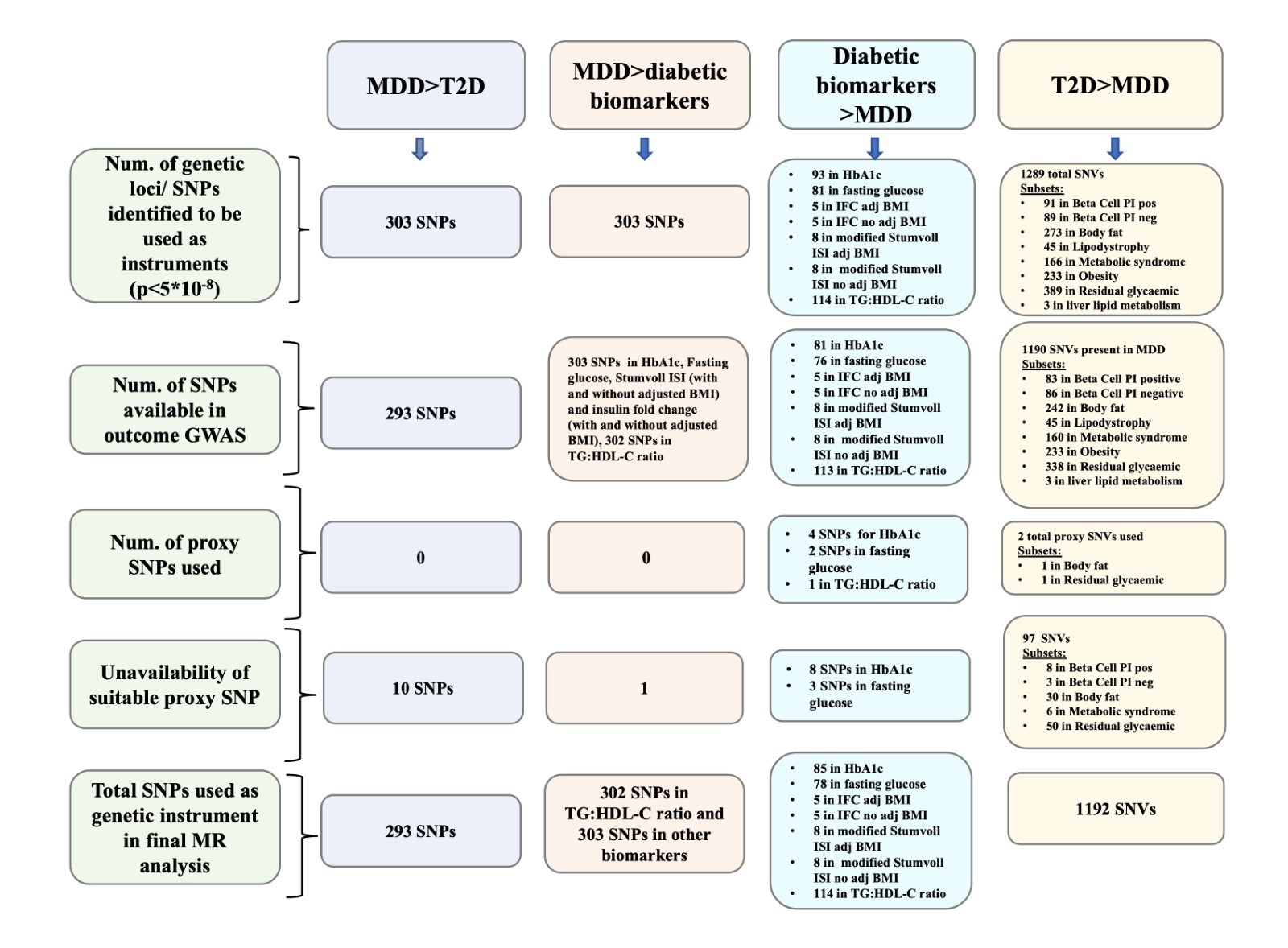


Fig S1: Details of genetic variants used as instruments, proxy SNPs and missing variants for each exposure group.  (Abbreviations used; T2D:  Type 2 Diabetes; MDD: Major depressive disorders; SNP: Single nucleotide polymorphism; MR: Mendelian randomization; ISI: Insulin sensitivity index; IFC: Insulin fold change; PI: proinsulin; pos: positive; neg: negative; BMI: Body mass index; SNV: single nucleotide variants; GWAS: Genome wide association study, TG-HDL-C: Triglycerides to high density lipoprotein cholesterol ratio, HbA1c: glycated haemoglobin).

 
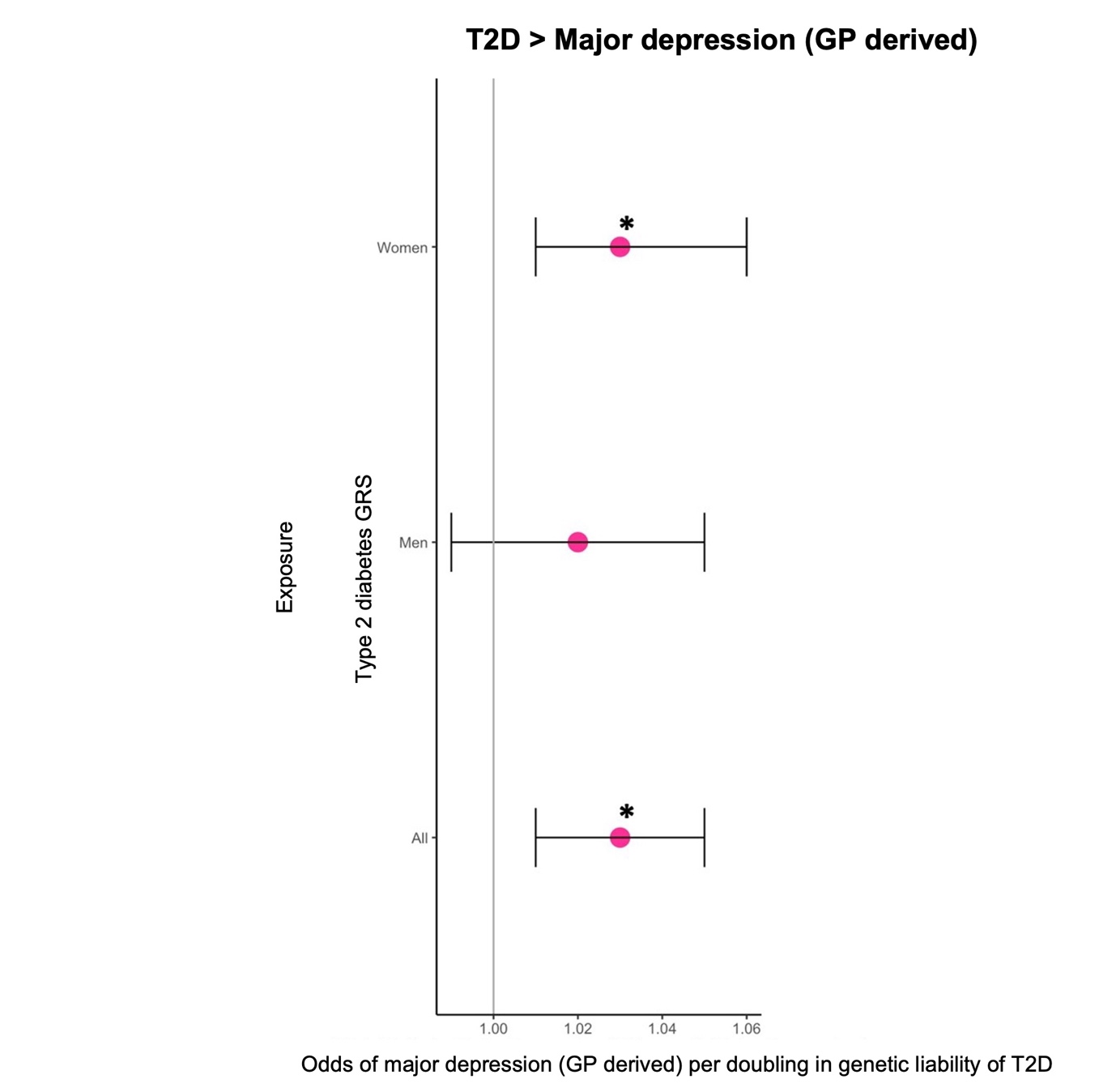


Fig S2:  1-sample UVMR in UK-biobank shows higher odds of major depression (GP derived) per doubling in T2D genetic liability using GRS in all individuals and women. *p value<0.05. (Abbreviations used: UVMR: univariable Mendelian randomization, GP: general practitioner, GRS: genetic risk score)

 
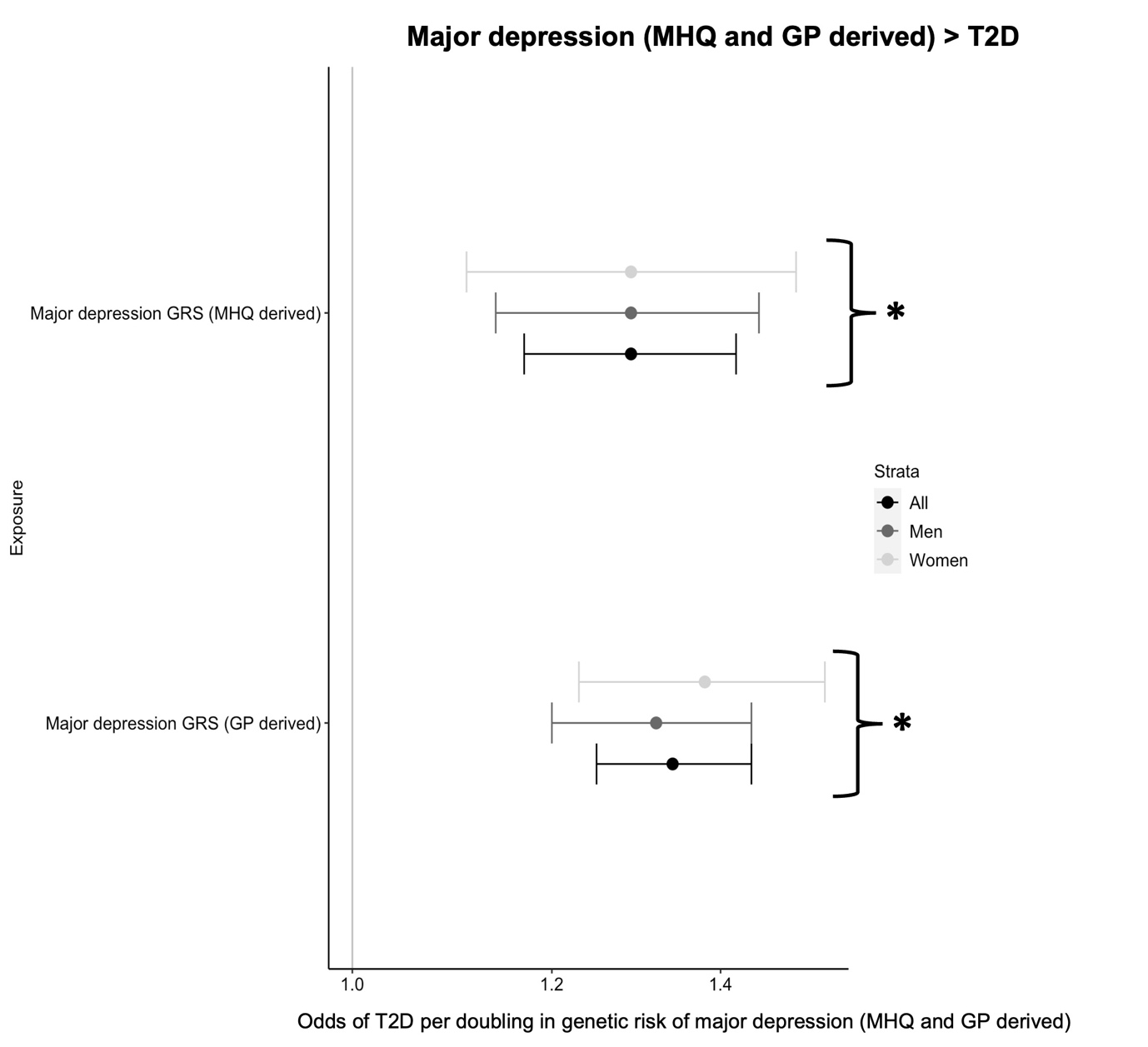


Fig S3: 1-sample UVMR in UK-biobank shows the higher odds of T2D per doubling in genetic risk of major depression (MHQ and GP derived) using GRS in sex stratified analysis. *p value <0.05 (Abbreviations used, UVMR: univariable Mendelian randomization, MHQ: mental health questionnaire, GP: general practitioner, GRS: genetic risk score).

 
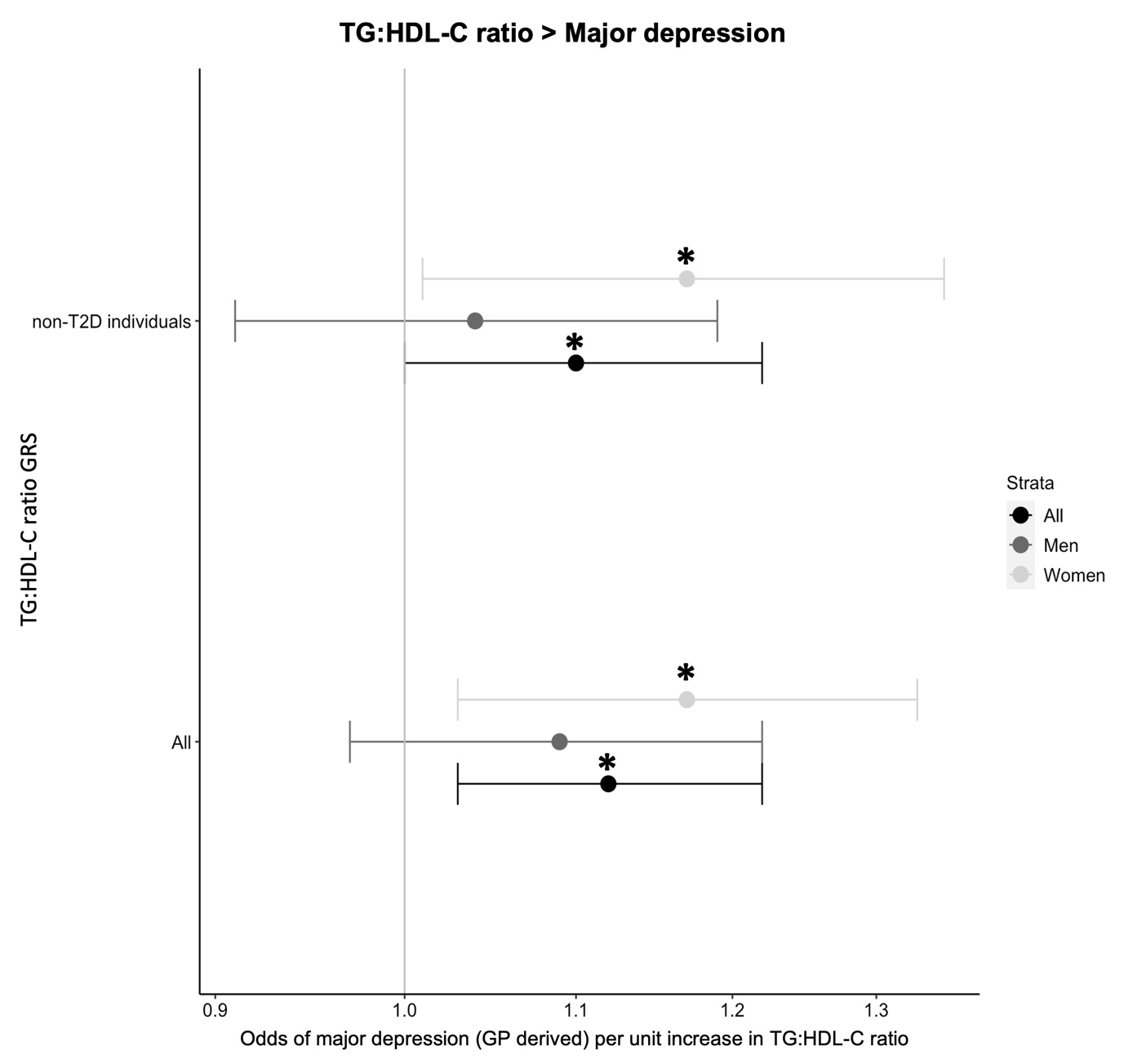


Fig S4: 1-sample UVMR in UK-biobank shows higher odds of major depression (GP derived) (in all individuals and non-T2D individuals) in sex stratified analysis per unit increase in TG:HDL-C ratio GRS. *p value <0.05 (Abbreviations used, UVMR: univariable Mendelian randomization, GP: general practitioner, GRS: genetic risk score, TG-HDL-C: Triglycerides to high density lipoprotein cholesterol ratio)

**Details of Supplementary tables:**

**ST 1:** Summary of genetic variants used as instruments for type 2 diabetes, **ST 2:** Summary of genetic variants used as instruments for major depressive disorder (MDD), **ST 3:** Details of genetic variants used as instruments for MDD (EAS vs. EAS ancestry) **ST 4:** Details of genetic variants used as instruments for various diabetic biomarkers (source: MAGIC consortium), **ST 5:** Summary of genetic variants available to be used as instruments for MDD and T2D, **ST 6:** 2-sample MR results represents the raw causal effect estimates and the odds of MDD risk, **ST 7:** 2-sample MR HORSE method in different exposure and outcome groups, **ST 8:** Results of MR LAP method demonstrating the evidence of potential bias from sample overlap, **ST 9:** 1-sample UVMR and MVMR (adjusted for BMI) in UK-Biobank, **ST 10**: 2-sample MR results represents the raw effect estimates and the odds of MDD in EAS, **ST 11:** 2-sample MR results for the odds of T2D and change in diabetic biomarkers, **ST 12:** 2-sample MR results representing effect estimates and odds of T2D across different ancestry groups, **ST 13:** 2-sample MR demonstrating the raw effect estimates and the odds of MDD
